# Feasibility of Real-time Behavior Monitoring Via Mobile Technology in Czech Adults Aged 50 Years and Above: 12-Week Study With Ecological Momentary Assessment

**DOI:** 10.2196/15220

**Published:** 2021-11-10

**Authors:** Steriani Elavsky, Adam Klocek, Lenka Knapova, Martina Smahelova, David Smahel, Richard Cimler, Jitka Kuhnova

**Affiliations:** 1 Department of Human Movement Studies University of Ostrava Ostrava Czech Republic; 2 Faculty of Social Studies Masaryk University Brno Czech Republic; 3 Faculty of Social Studies Masaryk University Interdisciplinary Research Team on Internet and Society Brno Czech Republic; 4 Faculty of Science University of Hradec Karlove Hradec Kralove Czech Republic

**Keywords:** mHealth, mobile phone, older adults, health behavior, physical activity, Fitbit

## Abstract

**Background:**

Czech older adults have lower rates of physical activity than the average population and lag behind in the use of digital technologies, compared with their peers from other European countries.

**Objective:**

This study aims to assess the feasibility of intensive behavior monitoring through technology in Czech adults aged ≥50 years.

**Methods:**

Participants (N=30; mean age 61.2 years, SD 6.8 years, range 50-74 years; 16/30, 53% male; 7/30, 23% retired) were monitored for 12 weeks while wearing a Fitbit Charge 2 monitor and completed three 8-day bursts of intensive data collection through surveys presented on a custom-made mobile app. Web-based surveys were also completed before and at the end of the 12-week period (along with poststudy focus groups) to evaluate participants’ perceptions of their experience in the study.

**Results:**

All 30 participants completed the study. Across the three 8-day bursts, participants completed 1454 out of 1744 (83% compliance rate) surveys administered 3 times per day on a pseudorandom schedule, 451 out of 559 (81% compliance rate) end-of-day surveys, and 736 episodes of self-reported planned physical activity (with 29/736, 3.9% of the reports initiated but returned without data). The overall rating of using the mobile app and Fitbit was above average (74.5 out of 100 on the System Usability Scale). The majority reported that the Fitbit (27/30, 90%) and mobile app (25/30, 83%) were easy to use and rated their experience positively (25/30, 83%). Focus groups revealed that some surveys were missed owing to notifications not being noticed or that participants needed a longer time window for survey completion. Some found wearing the monitor in hot weather or at night uncomfortable, but overall, participants were highly motivated to complete the surveys and be compliant with the study procedures.

**Conclusions:**

The use of a mobile survey app coupled with a wearable device appears feasible for use among Czech older adults. Participants in this study tolerated the intensive assessment schedule well, but lower compliance may be expected in studies of more diverse groups of older adults. Some difficulties were noted with the pairing and synchronization of devices on some types of smartphones, posing challenges for large-scale studies.

## Introduction

### Background

With an aging population, there is an increase in the prevalence of chronic conditions, functional limitations, and disability [1]. Although much of the age-related decline in health and functioning can be mitigated through healthy lifestyles [2], the prevalence of unhealthy lifestyle habits remains high globally as well as across the EU member countries. For example, one-third of Czech adults fall short of physical activity (PA) public health recommendations, with individuals aged 40-64 and >65 years being 1.7 and 4 times more likely to have low levels of PA compared with young adults, respectively [3]. Sedentary behavior is also prevalent among Czech adults [4,5], and nearly one-quarter of the Czech population reported experiencing sleep problems [6], suggesting an urgent need for developing effective behavioral interventions to improve the quality of life of Czech older adults.

Traditional intervention (face-to-face) approaches have been successful in promoting healthy behaviors (such as PA or sleep) [7,8] but require substantial resources and have limited public health impact. Advances in digital technology have opened up opportunities for delivering behavioral interventions in a way that is scalable and with potentially greater reach [9]. For example, there is evidence that mobile technologies, along with wearable devices, can be effective in increasing PA and reducing sedentary behavior in both healthy and clinical populations of adults [10-12]. At the same time, dynamic real-time ecological ambulatory methodologies (DREAM) [13] have been advocated for use in surveying behavioral risk factors, psychological states, social context, and various physiological parameters. In combination with digital technology platforms (eg, smartphones or tablets). These methods can also be used for the delivery of ecological momentary interventions delivered *just in time* and in people’s natural environments [14,15]. For these methods to be successfully used among older adults, it is critical to first obtain preliminary data on the feasibility of using technology-based methods (such as DREAM).

The evidence of implementing such approaches however comes mainly from the so-called *frontrunner* countries with a high level and fast rate of digital transformation [16]. Studies from countries with relatively low technological penetration (internet and mobile) are rare. Among such countries is the Czech Republic, where the digital gap between younger and older populations grows wider with age [17]. In particular, the penetration of mobile technology remains very low. Only 13% of Czech adults aged between 55 and 74 years report accessing the internet via a mobile phone in national data estimates (compared with a rate of 87% in those aged 18-24 years). The rate of use of mobile internet in the 55-74 years age group is also significantly lower than that in comparable countries such as Slovakia, Poland, and Hungary, where it ranges from 19% to 25% [18].

To date, only a few studies have focused on the feasibility of technology-based ecological momentary assessment (EMA) specifically in older adult populations, and the majority are also from the American context where the penetration of mobile technology is high. Ramsey et al [19] focused specifically on American older adults with cognitive and emotional difficulties and reported total EMA response rates of 46% and 48% for the two 10-day collection periods, respectively, with 70% of participants completing at least 30% of the surveys. The reasons for nonadherence ranged from being busy during the alarm, not hearing the alarm, to technical difficulties with the questionnaire or the phone. The authors further pointed out that supervised practice with the smartphone as well as more rapid technical assistance appeared to improve adherence rates. This rather low compliance stands in sharp contrast to the compliance rates reported by Fritz et al [20], who focused on community-dwelling older African Americans. The participants completed smartphone questionnaires (and provided saliva samples) 4 times per day for 7 consecutive days, with compliance for the individual variables ranging from 92% to 98%. The high compliance rates may be partially owing to the involvement of older adults in pilot-testing of the EMA interface and including a thorough, 4-step training protocol. Very high compliance (average adherence per person 86.4%) was also noted in a study of American older adults with HIV [21], which used a week-long EMA protocol with 5 prompts per day. Surprisingly, no participants reported difficulties in navigating the smartphone, but 40% reported that the research smartphone interfered with their activities slightly. Nonetheless, a recent study of EMA assessment of PA in American older adults found no evidence of interference with ongoing PA when answering EMA prompts 6 times per day across a 10-day study, with 92% of prompts being completed [22]. In that study, the older adult participants were also compliant with wearing of the ActivePAL activity monitor, with 73% of participants never removing the monitor. The study offered a prorated incentive of up to US $80 (for more than 80% EMA prompts answered).

A recent study by Liu and Lou [23] evaluated an EMA protocol to collect biopsychosocial data from community-dwelling older adults in China, a country with lower rates of mobile technology adoption than the United States. The study lasted one week, and participants completed six assessments per day, consisting of a short survey and a 30-second–long smartphone-based electrocardiogram recording. The total response rate was 91.5%, and younger women (50-59 years) showed the highest compliance (93.3%). The high level of compliance could be partially explained by the fact that while participants received random prompts during the 6 time intervals, they could complete the assessment at any time during these two-hour windows, even before receiving the prompt, as well as by the monetary incentive rewarded upon study completion (approximately US $60). Participants reported little difficulty with the EMA system and app but perceived carrying the research smartphone as inconvenient and the EMA prompts as interfering with their daily activities.

Importantly, all the mentioned EMA feasibility studies used research smartphones and did not make use of participant-owned devices. Although this strategy is useful in low-income populations or populations where smartphone ownership is low, it increases the financial burden for the researcher and might also pose issues for participants who are completely inexperienced with such technology. In addition, as noted by some older adults in previous studies, carrying an extra device (or two in the case of a connected sensory input device) may be perceived as inconvenient, especially when monitoring for longer periods (weeks or months). It could also be argued that the relatively high adherence rates reported in previous studies with older adults (with over 90% of studies reporting compliance rates above 80%) [24] may partly reflect the reality of carrying a *research-only* device that is novel or new and requires dedicated attention throughout the study. Lower adherence rates may be expected when data are collected through participant-owned smartphones due to the use of the device habitually and for multiple purposes. Nonetheless, such an approach may lead to more ecologically valid estimates of the EMA compliance rates. Together with data from culturally diverse samples (such as from countries with lower or slower rates of adoption of mobile technologies), this would help build more robust evidence of feasibility and more nuanced data on compliance and would generally help expand our knowledge of the dynamic processes (psychological and social) underlying health behaviors and technology use.

Therefore, we were interested in evaluating the feasibility of real-time behavior monitoring using participant-owned devices in a population with relatively low rates of smartphone penetration. In addition, as most previous feasibility studies involved relatively short study protocols (6-10 days), we were interested in evaluating the feasibility of repeated EMA data collection. This approach, sometimes referred to as the measurement burst design [25], is suitable for use in prospective longitudinal or intervention studies, where it is of interest to assess long-term trends along with short-term variability or effects of treatment on variability in outcomes of interest (such as was the case in the study by Ramsey et al [19]).

### Objective

This study aims to evaluate an EMA protocol administered repeatedly (in three separate bursts) through participant-owned smartphones and a connected Fitbit monitor to (1) evaluate the feasibility of prospective behavioral monitoring and (2) assess compliance rates and participant experience with intensive psychosocial data collection using a custom-made mobile survey app in adults aged 50 years and older.

## Methods

### Research Design

Our 12-week observational study used a mixed methods design, with longitudinal data collection via smartphones and fitness trackers to obtain three 8-day measurement bursts of data. Participants also completed web-based questionnaires (pre- and poststudy surveys) and participated in the focus groups. The participants received a prorated incentive of 600 CZK (US $27.41) for the study completion. All participants provided written informed consent before the study, and all study procedures were approved by the Ethics Committee of Masaryk University.

### Participants and Study Procedures

Participants (N=30; mean age 61.2 years, SD 6.8 years, range 50-74 years; 16/30, 53% male; 7/30, 23% retired) were recruited from the community (mainly the city of Brno and surrounding areas) through advertisements disseminated in both paper (leaflets) and electronic form (web-based posts on the study website, Facebook, or email advertisements), among organizations serving older adults (eg, libraries, senior clubs, and universities of *third* age) and subsequently through a chain-referral method. The recruited participants were representatives of 3 of the 13 regions in the Czech Republic (Southern Moravia–Brno, Northern Moravia–Ostrava, Opava, Bruntal, and Central Bohemia–Prague). They also represent 3 of the most populous regions (and the 3 largest cities in the Czech Republic–Prague, Brno, and Ostrava). The inclusion criteria were (1) age ≥50 years, (2) ownership of a smartphone with an Android operating system (version 5.0 and higher), (3) ability to connect to the internet (via Wi-Fi or a data plan), and (4) capability of normal PA (ie, having no contraindications to PA diagnosed by a medical doctor).

The participants provided informed consent and completed a web-based baseline questionnaire. Subsequently, they were invited to a personal orientation meeting where the study procedures were explained. At this meeting, participants received a Fitbit Charge 2 monitor, and the Fitbit app along with a survey app developed for the study were installed on their smartphones. The participants received instructions on the use of the monitor and the survey app. Owing to time and schedule constraints, some participants were unable to attend in-person group instruction sessions. Therefore, some participants were instructed in person individually at their time and place of preference (n=10), and some took care of the setup themselves based on email instructions and an instructional video provided by the researchers (n=5). At the end of the study, 3 focus groups took place, with a total of 15 participants (ie, 15/30, 50% of the sample).

### Measures

#### Feasibility and Study Experience

We assessed feasibility (ie, the practicality and ease of implementation of the study methodology) with a number of indicators across key aspects of the study.

#### Completion of Questionnaires

Two web-based questionnaires were administered as part of this study. The baseline web-based questionnaire (presurvey) collected basic demographic information (age, gender, education, and retirement/occupation) along with self-reported data on health status and medication use. Participants also completed a set of self-reported health behaviors and psychological measures [26-30]. As a measure of mobile health (mHealth) use, participants completed questions about the use of different information communication technology devices, including smartphones (frequency of use and duration). As a measure of smartphone literacy, we asked participants to rate their smartphone skills using a scale developed for the study. The 22-item scale assessed smartphone literacy across 3 areas (technical skills, communication, and security) using a 5-point Likert-type response scale and reflected digital literacy items from other existing tools/studies [31-39]. The internal consistency of the scale was good (Cronbach α=.938). The postsurvey contained a 10-item System Usability Scale [40] with instructions to rate the overall user experience with the smartphone, Fitbit, and survey mobile app. As additional measures of feasibility, we also included concrete questions on the perceived ease or difficulty of the smartphone/Fitbit/app setup, use, and comprehension of the study instructions. Also included was the same set of self-reported health behavior and psychological measures [26-30] as in the presurvey.

#### Focus Groups

We organized 2 focus groups with 15 participants each. Each focus group consisted of two 45-minute sections separated by a 15-minute snack break. The topics discussed were three areas: experience in the study, perceptions of future use of Fitbit devices, and the potential of Fitbit and similar devices in providing care (asked only in one of the 2 focus groups where participants were all caregivers). The focus group protocol is included in the Multimedia Appendix 1.

#### Compliance

We assessed compliance in two key aspects of the study.

##### The EMA

The EMA protocol was designed to provide snapshots of short-term variability in physical inactivity, sleep, selected psychological indicators, and context. The protocol included three 8-day bursts with 3 pseudorandom surveys, each sent during one of 3 preset time windows (8:00 AM-11:59 AM, 12:00 PM-3:59 PM, 4:00 PM-7:59 PM) and spaced a minimum of 90 minutes apart. Participants also completed an end-of-day report before bedtime (available from 8:00 PM-11:59 PM). In addition, participants were instructed to complete a brief report about any bouts (at least 10 minutes) of planned PA at moderate intensity or higher after each PA episode. This was done either through a self-initiated survey or as part of a *contextual* survey prompted by the server, when incoming Fitbit data indicated a bout of sustained PA lasting at least 10 minutes (with ±2-minute tolerance and threshold for activity set at >100 steps per minute). The custom-made EMA app also operated in an offline mode and sent a notification to signal an EMA prompt/questionnaire. Participants had 45 minutes to complete the questionnaire and were notified about an unfinished questionnaire every 5 minutes during this period with an option to snooze the questionnaire for 20 minutes. After 45 minutes from the initial signal, the questionnaire became inaccessible and was sent to the server (if connected to the internet or later when the connection was established) as it is, even if incomplete. The EMA protocol is described in the supplementary files.

##### The Fitbit Assessment

Participants wore the Fitbit Charge 2 monitors. Fitbit data were regularly (every 5 minutes) downloaded by a custom-made system from the Fitbit cloud and stored on a secure server where the data from psychosocial daily and momentary surveys were also stored for future analyses. All data gathered in this secure database allowed researchers to view, process, and evaluate the data throughout the study and were available on a web server for researcher access. The server also has an application programming protocol that enables access to data from other systems and scripts. Data retrieved from the Fitbit cloud included sleep parameters, heart rate (beats per minute), step count, and active minute data.

### Data Analysis

Descriptive statistics were computed using the statistical software IBM SPSS version 25 to describe the participants and compliance rates with the study protocol. Gender differences were analyzed using independent-sample *t* tests. Changes in self-reported behaviors and psychological outcomes from baseline to 12 weeks in the web-based pre- and postsurveys were assessed using a paired sample *t* test. Repeated measures analysis of variance was used to test the difference in PA in and out of assessment bursts. Qualitative data from the focus groups were transcribed verbatim and analyzed using thematic analysis [41].

## Results

### Sample Characteristics

The sample was balanced by gender (16/30, 53% male vs 14/30, 47% female), and the average age was 61.2 (SD 6.8) years. The majority of the sample was still actively working (22/30, 73%), and a slight majority had college education (56.7%), which is higher than the population average of 24% [42] and expected given that smartphone ownership increases with education level [43]. The participants used smartphones on average for about 3 years and rated their smartphone skills fairly highly (an average of 96 points on a 110-point scale). Most participants stated that they regularly accessed the internet on their smartphone (26/30, 87%) using a combination of Wi-Fi and a data plan (22/30, 73%). The reported use of health or fitness-related mobile apps before the study was relatively low, with 43% (13/30) of participants stating they never use them, 10% (3/30) stating they use them once per week or once per month, and 37% (11/30) stating they use them daily. The descriptive characteristics of the participants are presented in Multimedia Appendix 2, Table S1.

### Feasibility and Experience With the Study

All participants completed the pre- and poststudy surveys. Consistent with the observational nature of the study, there were no changes in self-reported parameters assessed in the web-based pre- and postsurveys, although 87% of participants stated that they were paying attention to the steps walked throughout the study.

The overall rating of using the mobile app and Fitbit was above average (74.5 out of 100 on the System Usability Scale) in the poststudy web-based survey. The majority reported that Fitbit (27/30, 90%) and the app (25/30, 83%) were easy to use and rated their experience positively (25/30, 83%). The participants indicated that the survey length and frequency were acceptable, and two-thirds stated that they could envision continuing data collection beyond the 12-week study (Multimedia Appendix 2, Table S2).

In terms of participant experience with the study protocol, focus groups revealed that some surveys were missed owing to notifications not being noticed or that participants would need a longer time window for survey completion. Some participants mentioned that they would prefer receiving *confirmation of receipt* of the completed surveys and perceived that contextual PA questionnaires were not triggered as they should be (ie, the survey did not always come automatically after an episode of what participants perceived as *moderate* PA). In terms of the Fitbit monitor, participants expressed an interest in knowing how the monitor works/measures activity. Some found wearing the monitor in hot weather or at night uncomfortable, noting technical issues such as the display not being visible in direct sunlight or display disturbing sleep during the night by lighting. Some participants voiced concerns over the imprecision of sleep measurement and wished the Fitbit app (which they were not required to use during the study) were available in the Czech Republic. One of the 3 participants who experienced synchronization issues between their smartphone and the Fitbit monitor noted this issue in the focus group. Overall, participants were, however, highly motivated to complete the surveys and to be compliant with the study procedures.

Regarding the potential of similar mobile technologies and their future use, the focus group participants placed high value on being able to self-monitor their behavior (especially steps and sleep) and receive personalized feedback. They expressed interest in motivational components being incorporated and using these tools as part of personalized interventions. Some mentioned the desire to share their data with their physician and obtain additional insights into their health and habits, although a number of challenges with regard to this were noted, ranging from perceived lack of time on the side of physicians, lack of motivation on both the patient and physician side, or technical issues involved in setting up such a monitoring system.

### Compliance

#### Compliance With Long-term Monitoring Via EMA

All 30 participants completed the study. Across the three 8-day bursts, the participants completed 1568 out of 1906 (82.3% compliance rate) surveys administered 3 times per day at a pseudorandom schedule, 481 out of 613 end-of-day surveys (78.5% compliance rate), and 736 episodes of self-reported planned PA (with 29/736, 3.9% of the reports being initiated but returned without data). The survey completion rates for the three bursts are presented in Multimedia Appendix 2, Table S3.

**Figure 1:**
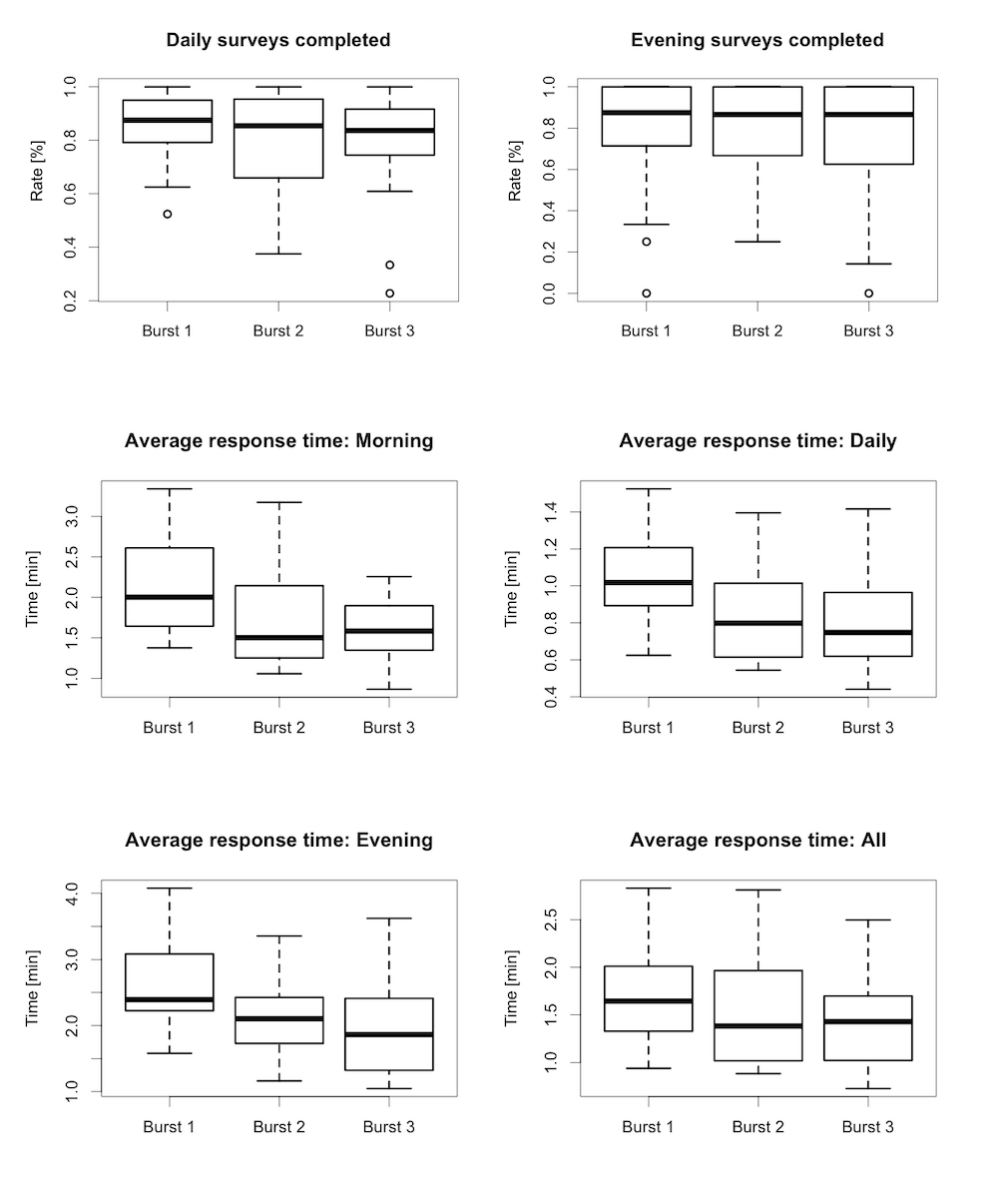
Completion rates (%, top row) and survey response times (minutes, bottom two rows) by survey and burst.

**Figure 2:**
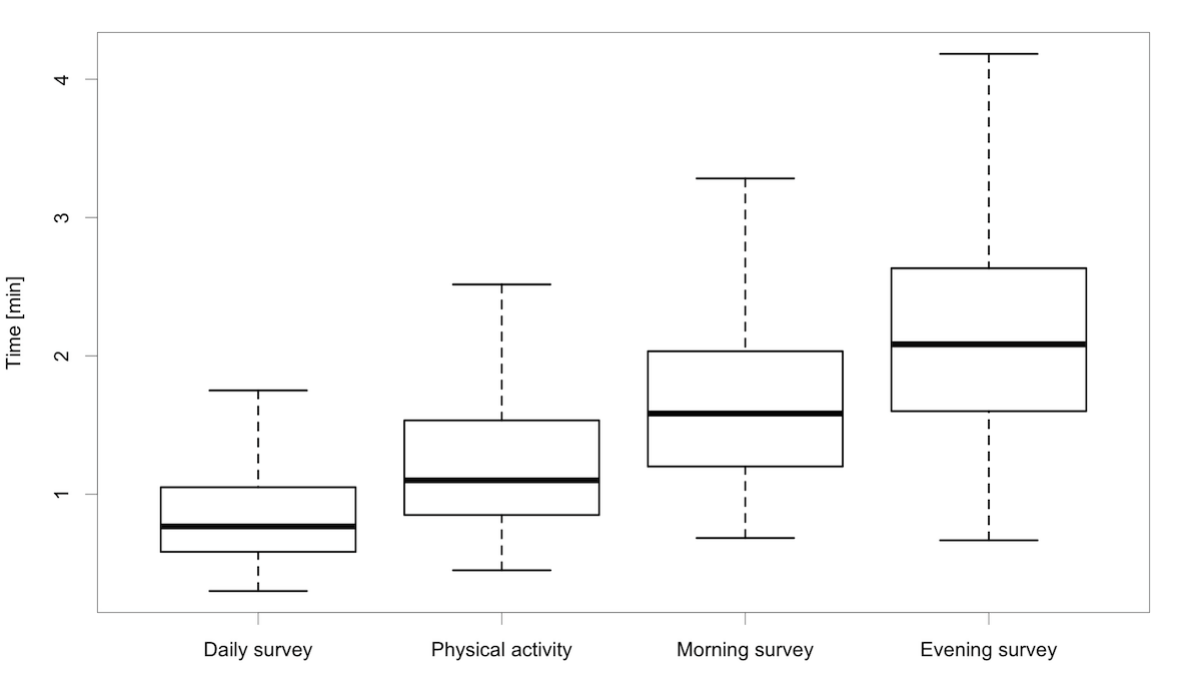
Overall survey response times by survey.

The average duration of completion for each type of survey is presented in Multimedia Appendix 2, Table S3, along with the total response times for each type of survey and across the three bursts of intensive monitoring (Figures 1 and 2).

#### Compliance With Long-term Monitoring Via Fitbit

In terms of Fitbit compliance, upon enrollment, 3 participants had problems with syncing their phones with the Fitbit devices (primarily the lite version of HUAWEI smartphones) and had to use a backup device (computer or tablet) for syncing. On average, participants wore the Fitbit for 20.58 (SD 3.93) hours per day, with 20 participants having no missing days. A total of 3 participants had ≥30 days of Fitbit data missing across the 12 weeks, and this was due to synchronization problems that were not resolved in time and led to loss of data (Multimedia Appendix 2, Table S4).

**Figure 3:**
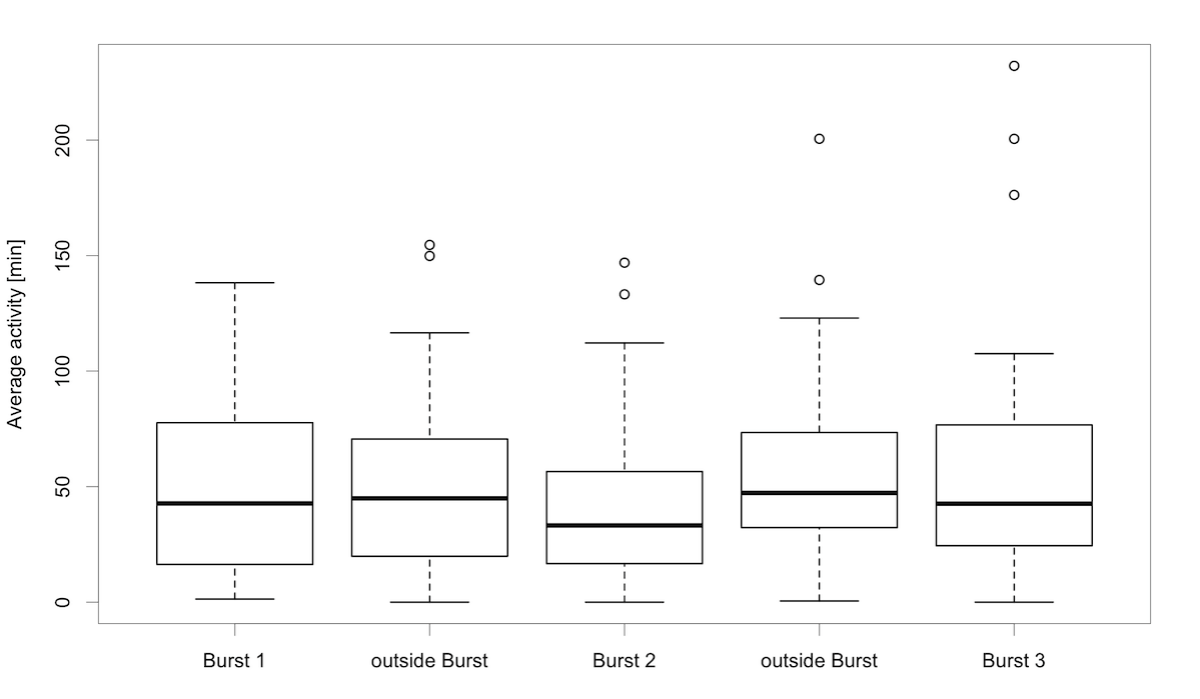
Average moderate intensity physical activity (in minutes) across bursts.

**Figure 4:**
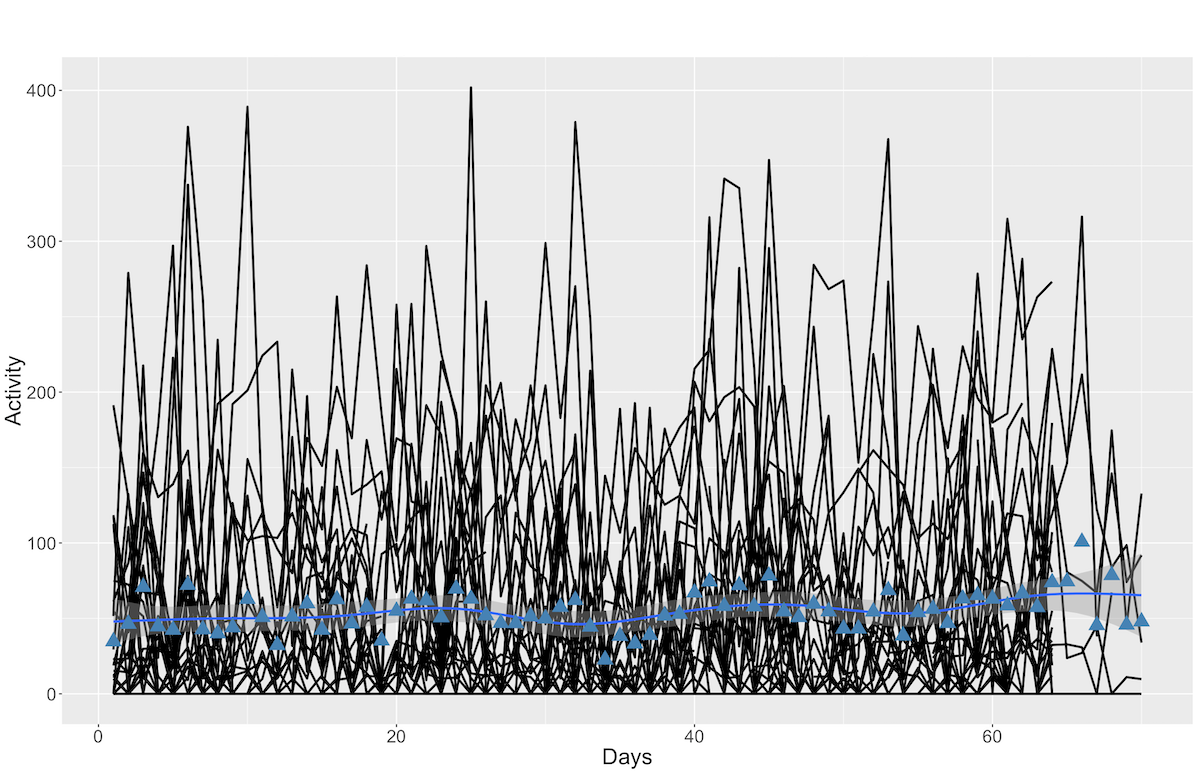
Continuous moderate intensity physical activity (in minutes) across the study. The blue line represents the sample average across the study. The black lines represent person-level variability in physical activity.

Analysis of Fitbit data indicated no differences in measured PA between bursts or periods outside of measurement bursts (*F*_4,131_=0.624; *P*=.65; Figure 3), but there was substantial within-person variability in PA across the 12-week monitoring period (Figures 3 and 4).

## Discussion

### Principal Findings

This study demonstrated the feasibility of intensive behavior monitoring via mobile technology in adults aged ≥50 years, in a research design where (unlike in previous studies [22,23]) participants’ own smartphones were used. The participants found the system of using a Bluetooth-connected fitness bracelet along with daily surveys presented through an app on their own personal smartphone as easy to use and were not overly bothered by the frequency and length of the surveys. This is encouraging, as it suggests the potential for reducing both financial burden on the side of the researchers as well as participant burden in carrying more than one device. Participants were compliant (79%-82%) with the study protocol across the 12-week study duration with acceptable survey completion rates across all three 8-day measurement bursts. Preliminary analysis of Fitbit data did not suggest meaningful reactivity effects to the monitoring, as overall PA levels appeared stable between burst and out-of-burst periods (Figure 4). These findings demonstrate the feasibility of using similar protocols in the context of longitudinal or intervention studies targeting older adults, even in cultural contexts where overall rates of smartphone ownership and experience with mHealth tools are low.

### Comparison With Prior Work

Reviews of previous research with older adults [10-12] demonstrate that the majority of existing studies included participant samples from mostly frontrunner countries in terms of digital technology adoption, whereas the Czech Republic represents a population that currently trails behind in their use of mobile technologies to support healthy lifestyles compared with their European counterparts [18,44]. Previous feasibility studies in older adult populations also incorporated short EMA protocols (lasting 6-10 days). One study that collected EMA data in more than one burst (two 10-day periods before and after an intervention for individuals with cognitive and emotional difficulties) showed rapidly lower compliance rates (46%-48% adherence for all surveys) [20] than studies with only one EMA collection period only (86.4%-98%) [20,22,23]. This study used a measurement burst design with three 8-day bursts of EMA data collection, with two-third of the participants indicating they would be willing to continue with the study protocol beyond the 12-week study duration. This may be partly owing to the fact that this study used the participants’ own smartphones. Previous studies have shown that a significant number of participants perceived carrying a *research* smartphone as inconvenient and interfering with their daily activities [23,45]. The results of this study show that this might be less of the case when relying on participants’ own smartphones. Although an EMA protocol with multiple random prompts during the day might still require participants to pay closer attention to their smartphones than they would on a regular day.

Although compliance was satisfactory overall, in this study, women had slightly higher response rates to the timed daily surveys compared with males (data not shown). The differences did not reach statistical significance but may reflect a commonly reported gender response bias that is reflective of a number of factors, including differences in motivation [46], rather than a survey mode of delivery (ie, smartphone).

### Implications

Overall, we conclude that it is feasible to incorporate repeated EMA assessments in larger prospective studies or as a method for evaluating the impact of interventions. Indeed, it was encouraging that many participants expressed an interest in receiving prompts and intervention features as part of the monitoring, boding well for the implementation of *just-in-time* PA interventions in this age group [14,25]. The successful implementation of mobile technologies in lifestyle promotion is critical to increasing the reach and public health impact of behavior modification and health promotion programs. Mobile technologies along with wearable devices have been found to be effective in increasing PA and reducing sedentary behavior, although larger studies with more rigorous methodologies are urgently needed [10-12].

The custom server in our study was built with the capability of accessing data from other systems and scripts via application programming protocol, which opens opportunities for monitoring other behaviors or bodily functions via technology and using these data to develop effective lifestyle or disease management interventions. Such efforts are also under way in the Czech Republic, where the first Czech National eHealth Center was established in 2012 with a focus on telemedicine. Nonetheless, this stands in stark contrast to the rather lukewarm attitude toward embracing such technologies among health care providers in the Czech Republic who remain reluctant to use mHealth technologies with older adults [47]. Participants in our focus groups expressed similar sentiments when discussing the potential of behavior monitoring as part of preventive health care. Clearly, more work is needed to develop sustainable protocols and systems for incorporating mHealth tools within existing health care infrastructures.

From a research standpoint, there are several lessons to be imparted from this study in terms of practical suggestions and the study design. First, researchers must allocate sufficient time to set up the devices and explain the study protocol. In this study, participants were capable of setting up the devices and apps on their own when they were unable to attend the group training sessions. Nonetheless, we recommend that any training session be followed by practice days to ensure sufficient familiarization with mobile apps, devices, and EMA prompts. When using a commercial device such as a fitness bracelet, researchers should be prepared to confront synchronization issues and have appropriate backup plans. In non–English-speaking countries, an additional challenge is presented when used apps are not in the native language of the participants, which is particularly relevant for older adult participants. In this study, we did not require any interaction with the Fitbit app; in fact, participants were asked not to use it. However, many were interested and explored the features on their own and expressed regret that the app was not translated to Czech (the Czech version became available only after this study). Finally, the participants in our study were eager to receive feedback at the end of the study. We provided detailed personalized feedback at the end of the study and informed the participants that they would receive it. However, many were pleasantly surprised at the level of detail and insights available and mentioned that they would have been more compliant and attentive to the data collection if they knew what type of feedback was possible. Researchers may want to provide examples of feedback reports at the beginning of the study as a way to increase motivation to comply with the study protocol.

### Limitations and Future Research

This was the first study using intensive behavior monitoring and survey data collection via mobile technology with Czech older adults and the first study to report the feasibility of relying on participants’ own smartphones when delivering an EMA study protocol. Although this may currently exclude some older adults who do not own a smartphone, this may be less of an issue in the future, given the rapidly rising numbers of smartphone users and older adults quickly catching up [43]. The sample was small, but there was variability across age, education, weight, and PA status (approximately half of the sample comprised physically active individuals). However, the study’s inclusion criteria (smartphone with Android, capable of normal PA) and recruitment strategy may have promoted the participation of younger individuals who are more likely to use technology.

We used an affordable, commercially available fitness tracker that measures PA with acceptable accuracy [48], but the same device was found to be less accurate in the staging of sleep compared with a medical device [49]. Future studies should carefully weigh the advantages and disadvantages of using research grade as opposed to commercial devices, with implications for measurement precision as well as participant interest and burden. Participants in our study enjoyed the feedback provided by the bracelet on its display, but this type of feedback may be undesirable in clinical or randomized controlled trials targeting PA. Using a combination of a research-grade device (for dependent outcome assessment) and a commercial device (as an intervention tool) has been suggested as a possible solution [50].

The system as a whole was rated well by the participants; however, technical difficulties were noted throughout the study. On the side of the participants, there were issues with the compatibility of some of their smartphones with the Fitbit device, resulting in 3 participants having to synchronize their Fitbit through a *third* device (eg, a laptop). Participants missed some surveys due to notifications not being noticed, further highlighting the need to conduct comprehensive pilot-testing and to develop effective training protocols before embarking on a full-scale study. The data provided by this study also served to fine-tune the data collection protocol and improve the robustness of the server/system solution for data integration. Future research and development are also focused on the creation of automatic reports from the server for researchers to obtain information about the current status of all participants (daily, weekly, and monthly) and the status of the ongoing data collection (eg, synchronization status of devices, battery levels, and questionnaire completion rate). Future development will also involve the integration of various streams of data and enhancement of dynamic features, including machine learning, to detect the most appropriate/opportune times to generate EMA prompts and dynamically select the most appropriate items for prompted surveys.

### Conclusions

The use of a wearable device coupled with a mobile survey app is feasible for use with older adults, who also indicate a high level of interest in motivational prompting and intervention components. Commercially available tools such as Fitbit devices offer a practical solution for both behavior monitoring and interventions in both small- and large-scale studies. The implementation of intensive measurement protocols, such as in this study, offers unique opportunities for insight into behavior dynamics (both short and long term).

## Appendix

Multimedia Appendix 1 Focus group protocol.

Multimedia Appendix 2 Ecological momentary assessment protocol.

## Abbreviations

EMA: ecological momentary assessment
DREAM: dynamic real-time ecological ambulatory methodologies
mHealth: mobile health
PA: physical activity
