# Supplementary material for: Feasibility of Real-time Behavior Monitoring Via Mobile Technology in Czech Adults Aged 50 Years and Above: 12-Week Study With Ecological Momentary Assessment
Source: JMIR Aging. 2021 Nov 10;4(4):e15220. doi: 10.2196/15220 (PMC8663589; doi:10.2196/15220)
Supplement: Multimedia Appendix 1 [file aging_v4i4e15220_app1.docx]

**FOCUS GROUP PROTOCOL – mHealth Active**

***Focus Group Format***

- Welcome + introduction (introduction of discussion leaders and introduction of roles)
- Distribution of name tags and markers
- Explanation of the intention/study goals
  - Why we meet
  - Form of the meeting - duration and content
    - two parts of 45 minutes, break, refreshments
- Explanation of technical aspects
  - Recording, anonymity and the like.
  - Review of the informed consent they have already signed (mainly the GDPR page).
- Duration of the meeting:
  - 2 x 45 minutes – in-between break of 15 min max for small snacks (biscuits, coffee, tea water), toilet break (before the break explain where the toilet is).
- Recording on camera (not pointed at the face)
  - Just in case - backup for transcripts - so we know who's talking. Continuously address respondents by name - to facilitate transcripts.
- Audio recording on dictaphones (recorded on two separate devices in case one fails).
- Sitting in a circle
  - pre-arranged room 2.53, in case of occupancy, room 2.61 is also possible (there is a small table on which there can be a dictaphone and water).
- Have at least two bracelets (Fitbits) available - so that they can look at them and show any problems.

***Focus Group Topics***

**Experience**

- *experience is also partially assessed in a post-study online questionnaire)*
- *focus on aspects not stated in the questionnaire*

„At the end of the study, you completed online questionnaire where you provided some feedback on your experience with the study. Now I would like to ask you to provide additional comments on any aspect of your experience:“

- Fitbit watch
  - Wear (also during sleep), data/step tracking
- Fitbit mobile app
- mHealth Active (survey) mobile app
  - frequency, length of surveys
  - problems with the app
- Overall experience in the study

Prompts: Can you think of any feedback? Something you didn't mention in the questionnaire, or the questionnaire didn't ask, but you find it important?

**Future Use**

- How do you perceive the future of the use of these devices for people your age in general?
- Under what conditions, for what purposes would you be willing to use Fitbit or a similar device in the future?
  - If few examples or very monotonous answers prompt more:
    - What about various interventions aimed at changing behavior? (e.g., more exercise, adjusting habits, quality of sleep, stress, adherence to taking medication on time, managing chronic diseases - diabetes, prescription – smoking cessation , depression management), or as prevention against diseases (obesity, cardiovascular disease, diabetes)?
  - If someone does not want to use the device - ask about barriers and what prevents them from using Fitbit or another device
- In your opinion, what could prevent people your age from using these interventions or preventive practices through cell phones and Fitbit bracelets or similar devices?
  - At the level of personal, social and systemic barriers - if they do not address each level, you can prompt for feedback on the topic.
- Can you imagine that the use of Fitbit and similar devices could become part of health care - that it would be possible to use them at a general practitioner who would prescribe them as part of prevention / intervention according to your needs and your state of health? This means that, for example, the doctor would have data from the application you are using and will incorporate information from it into medical treatment or procedures, for example.

(This question is omitted for caregivers).

**Caregivers**

„This group is unique in that everyone (you) takes care of another person, as you stated in the questionnaire. Is that so?“

„We would now focus on the care you provide to your a close person (family,friend or neighbor). You do not have to reveal any personal information or anything about the care that you would find unpleasant. We would mainly like to know if it is conceivable for you that a device like Fitbit or a similar device could be useful in the care that you provide and how.

- - The example uses could be similar interventions and preventive practices, as we have already mentioned. But additionally also for monitoring movement and mobility problems of caregivers using GPS, well-being of caregivers or care recipients, or communication with a doctor who would, for example, have access to the outputs of the application and the Fitbit bracelet. Further prompts:
    - Under what conditions?
    - For what purposes do you imagine that you could use it?
    - What prevents you from using it?
