# Supplementary material for: Feasibility of Real-time Behavior Monitoring Via Mobile Technology in Czech Adults Aged 50 Years and Above: 12-Week Study With Ecological Momentary Assessment
Source: JMIR Aging. 2021 Nov 10;4(4):e15220. doi: 10.2196/15220 (PMC8663589; doi:10.2196/15220)
Supplement: Multimedia Appendix 2 [file aging_v4i4e15220_app2.docx]

**Ecological Momentary Assessment (EMA) Study Protocol**

**Participants:** N=30

**Inclusion criteria:** 50+, owns smartphone with Android (min. version 5.0 or higher), capable of normal physical activity (i.e., without physical activity limitations diagnosed by a physician)

**Design:** 12-week observational study with a measurement burst design (3 x 8 days with 1 month apart)

**EMA protocol:** 4 x day (pseudo)random survey; plus self-initiated physical activity report; plus context-triggered physical activity survey

[Maximal number of timed surveys per person = 4 x 8 x 3 = 96; i.e. 2 880 data points for sample of n=30].

**EMA surveys:**

1. Timed trigger – 4 x day with a 90min buffer; one survey at random in predefined time windows:
   1. 8-11:59
   2. 12:00-15:59
   3. 16:00-19:59
   4. 20:00-22:00

Survey notification can be snoozed for 30 min with a notification reminder presented every 15 min. Survey is send to the server it is completed or after time window expires (even when not completed).

Notification windows for the morning and evening survey can be adjusted to adapt to individual wake or bed times.

1. Self-initiated trigger (physical activity report) – participant may report any episode of planned physical activity lasting at least 10 min at moderate intensity (defined as activity during which elevated heart rate and breathing rate occur) lasting 10 min or longer in the app by clicking on the physical activity report icon.
2. Contextual trigger – physical aktivity survey is triggered automatically when Fitbit registers physical activity of moderate intensity (defined as ≥100 steps per minute) for duration of 10 minutes or longer (+2 min tolerance). Survey can be snoozed for 30 min with a notification reminder presented every 15 min. Maximum of 3 triggered surveys are sent in one day.

**EMA training:**


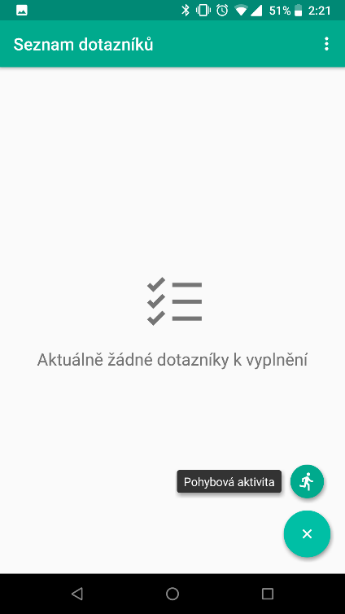


*Physical activity*

*Currently, no new surveys to complete*

English caption:

*List of surveys*


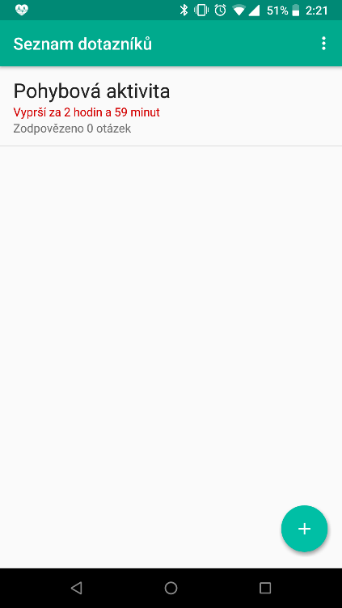

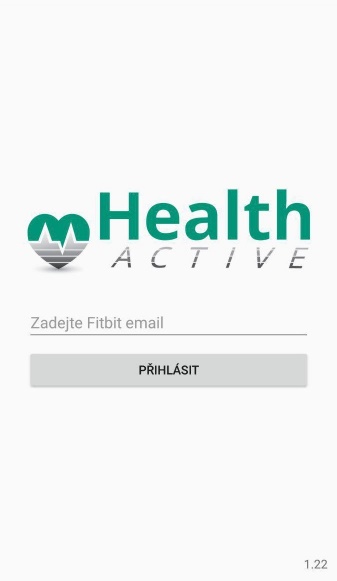


English caption:

*List of surveys*

*Physical activity*

*Expires in 2 hours and 59 minutes*

*Answered 0 questions*

English caption:

*Please enter your Fitbit email*

*Sign-In*

**
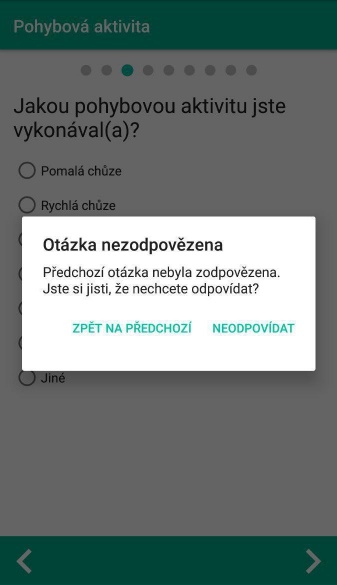

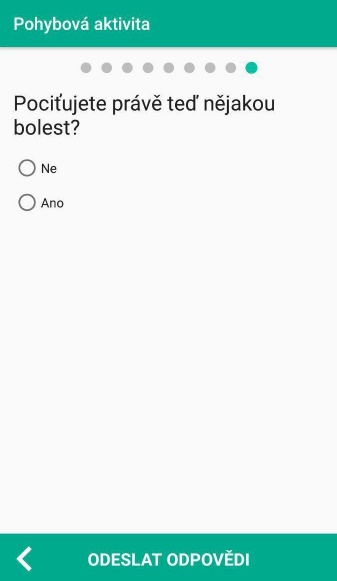

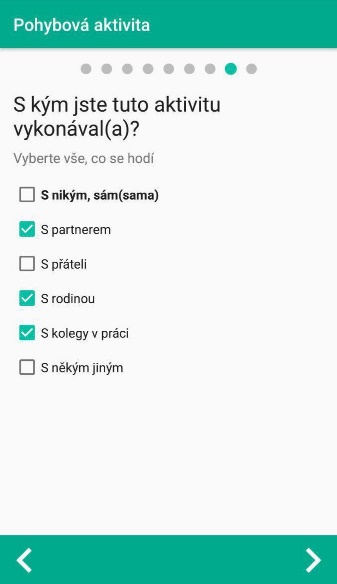

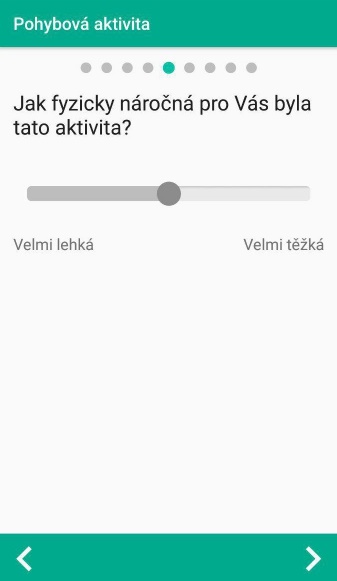
**

English caption:

In the background:

*What type of physical activity did you engage in?*

*Slow walk*

*Fast walk*

At the forefront:

*This question has not been answered*

*Previous question has not been answered. Are you sure that you do not want to answer it?*

*Back to previous Not answering*

English caption:

*Do you experience any pain right now?*

*No*

*Yes*

*Submit answers*

English caption:

*Who were you with when doing this activity?*

Select all that apply

*With no one, alone*

*With my partner*

*With friends*

*With family*

*With colleagues from work*

*With someone else*

English caption:

*How physically demanding was this activity for you?*

*Very light……………Very intense*

**TIMED SURVEYS**

**Morning (8-11:59) / Daily Surveys (12:00-15:59; 16:00-19:59)**

| **Item Order#** | **Variable** | **Item** | **Response Format** | **Branching** |
| --- | --- | --- | --- | --- |
| **Sleep** | | |  |  |
| **1.** | spa_1 | What time did you go to sleep yesterday night? | HOD:MIN (00:00) |  |
| **2.** | spa_2 | What time did you wake up this morning? | HOD:MIN (00:00) |  |
| **3.** | spa_3 | How many hours of sleep do you think you got last night? | Number of hours (scrolled) |  |
| **4.** | spa_4 | How would you rate the quality of your sleep last night? | (0) “Very poor” – (100) “Very good” (slider) |  |
| **Affect (random selection of one of two emotions)** | | |  |  |
| *Right now I feel*… | | | *Slider with word anchors* |  |
| **5.** | em_1 | Happy – Energetic | (0) “Not all” – (100) “Very much” |  |
| **6.** | em_2 | Calm – Content | (0) “Not all” – (100) “Very much” |  |
| **7.** | em_3 | Sad – Lonely | (0) “Not all” – (100) “Very much” |  |
| **8.** | em_4 | Nervous - Angry | (0) “Not all” – (100) “Very much” |  |
| **Daily stressors** | | |  |  |
| *In the past hour, were you bothered or stressed by any or the following situations? (select all that apply)* | | | *Checkbox* |  |
| **9.** | stres_1 | Problem with family, friends or other close persons | Select |  |
| **10.** | stres_2 | Problems with other people | Select |  |
| **11.** | stres_3 | Having too much to do and not enough time to do it | Select |  |
| **12.** | stres_4 | Having to solve something new and not knowing how | Select |  |
| **13.** | stres_5 | Feeling discriminated against because of race, gender, age of health status | Select |  |
| **14.** | stres_6 | I feel stressed for other reasons | Select |  |
| **15.** | stres_7 | *Nothing bothered or stressed me. | Select |  |
| **Daily stressors - intensity (branching IF stres1-10=YES (selected)** | | |  |  |
| *How much were you bothered or stressed by …* | | | *Slider with word anchors* |  |
| **16.** | stres_1a | Problem with family, friends or other close persons | (0) “Not all” – (100) “Very much” | **IF stres_1=YES** |
| **17.** | stres_2a | Problems with other people | (0) “Not all” – (100) “Very much” | **IF stres_2=YES** |
| **18.** | stres _3a | Having too much to do and not enough time to do it | (0) “Not all” – (100) “Very much”” | **IF stres_3=YES** |
| **19.** | stres _4a | Having to solve something new and not knowing how | (0) “Not all” – (100) “Very much”” | **IF stres_4=YES** |
| **20.** | stres_5a | Feeling discriminated against because of race, gender, age of health status | (0) “Not all” – (100) “Very much” | **IF stres_5=YES** |
| **21.** | stres_6a | I feel stressed for other reasons | (0) “Not all” – (100) “Very much” | **IF stres_6=YES** |
| **Context** | | |  |  |
|  | | | *Checkbox* |  |
| **22.** | kontx_1 | Where are you now? (select all that applies). | (1)at home, (2)outside, (3)a work, (4)at an organized activity (cinema, course, etc.), (5)other |  |
| **23.** | kontx_2 | Who are you with now? (select all applies). | (1)*Alone, (2)with close persons or friends(3)with strangers |  |
| **24.** | kontx_3 | What are you doing right now? (select all that applies). | (1)relaxing, (2)working,(3)having fun, (4)learning,(5)running errands,(6)other |  |

| **Plans for physical activity** | | |  |  |
| --- | --- | --- | --- | --- |
|  | | |  |  |
| **25.** | pl_1 | Do you intend to be physically active in the next 2-3 hours? | (0)“NO”, (1)“YES” |  |
|  |  |  | *Slider with word anchors* |  |
| **26.** | se_1 | How confident are you that you will be capable of engaging in physical activity that increases heart rate for at least 10 min straight in the next 2-3 hours? | (0) “Not at all (0% confidence)” – (100) “Completely (100% confidence)” | **IF pl_1=YES** |

**Note.** Questions spa_1-spa_4 appear only in the morning survey.*marks exklusive answer options

I.**TIMED SURVEYS**

**Evening (20:00-22:00)**

| **Item order#** | **Variable** | **Item** | **Response Format** | **Branching** |
| --- | --- | --- | --- | --- |
| **HRQL – Health-related quality of life** | | |  |  |
|  | | | *Slider with word anchors* |  |
| **1.** | hrql_1 | Today my energy level was… | (0) “Low” – (100) “High” |  |
| **2.** | hrql_2 | Today my physical health was… | (0) “Very poor” – (100) “Very good” |  |
| **3.** | hrql_3 | Today my mental health was… | (0) “Very poor” – (100) “Very good” |  |
| **4.** | hrql_4 | Today my physical health made it more difficult to do daily activities. | (0) “Not all” – (100) “Very much” |  |
| **5.** | hrql_5 | Today my mental health made it more difficult to do daily activities. | (0) “Not all” – (100) “Very much” |  |
| **6.** | hrql_6 | Today pain made more difficult to do my daily activities. | (0) “Not all” – (100) “Very much” |  |
| **7.** | hrql_7 | Today fatigue made more difficult to do my daily activities. | (0) “Not all” – (100) “Very much” |  |
| **Life satisfaction – quality of life** | | |  |  |
|  |  |  | *Slider with word anchors* |  |
| **8.** | qol_1 | Today, in most ways my life is close to my ideal. | (0) “Disagree” – (100) “Agree” |  |
| **9.** | qol_2 | Today,I am satisfied with my life. | (0) “Disagree” – (100) “Agree” |  |
| **Need for cognitive closure** | | |  |  |
| **10.** | nfcc_1 | Today my day had a clear and firm order. | (0) NO, (1) YES |  |
| **11.** | nfcc_2 | Today I had to face an unpredictable situation or solve a new problem. | (0) NO, (1) YES |  |
| **12.** | nfcc_3 | I made an important decision today. | (0) NO, (1) YES |  |
| **13.** | nfcc_4 | Today I was in an uncertain situation or faced an event that I did not understand. | (0) NO, (1) YES |  |
|  |  |  | *Slider with word anchors* |  |
| **14.** | nfcc_1a | Having a clear and firm order of the day suited me today. | (0) “Disagree” – (100) “Agree” | **IF nfcc_1=YES** |
| **15.** | nfcc_2a | Being in this situation was uncomfortable for me. | (0) “Disagree” – (100) “Agree” | **IF nfcc_2=YES** |
| **16.** | nfcc_3a | When making a decision today, I tried to solve everything as quickly as possible. | (0) “Disagree” – (100) “Agree” | **IF nfcc_3=YES** |
| **17.** | nfcc_3b | When making a decision today, I tried to consider different perspectives. | (0) “Disagree” – (100) “Agree” | **IF nfcc_3=YES** |
| **18.** | nfcc_4a | Being in this situation was uncomfortable for me. | (0) “Disagree” – (100) “Agree” | **IF nfcc_4=YES** |
| **Stres/Resilience** | | |  |  |
|  |  |  | *Slider with word anchors* |  |
| **19.** | stresd_1 | How stressful was today for you overall? | (0) “Not at all” – (100) “Very much” |  |
| **20.** | res_1 | Today I coped with stressful situations … | (0) “Very poorly” – (100) “Very well” |  |
| **Context/Physical activity** | | |  |  |
|  |  |  | *Numerická škála* |  |
| **21.** | kontxd_1 | How typical was today for you? | 1. “ Same as usual” 2. “A little different than usual” 3. “Very different than usual” |  |
| **22.** | kontxd _2 | Have you attempted to exercise or be physically active today? | (0)“NO”, (1)“YES” |  |
| **23.** | ss _1 | To what extent did you feel supported by your surroundings (friends or family) to be physically active today? | (0)“Not at all” – (100) “Very much” | **IF kontxd_2=YES** |
| **24.** | ba_1 | Did anything make it difficult or stop you from exercising or doing physical activity today? (select the main obstacle) | (1)*Nothing, (2)lack of time, (3)weather, (4) lack of motivation,(5)pain/injury/illness, (6)don´t know how, (7)other | **IF kontxd_2=YES** |
| **25.** | ba_1a | What else prevented you from exercising or doing physical activity today? | Text answer | **IF ba_1=7** |
| **26.** | fa_1 | What made exercise or physical activity easier for you today? (select the main helpful thing) | (1)*Nothing, (2)exercise buddy, (3)support from friends (4)support from family (5)fitness bracelet or mobile app, (6) other | **IF kontxd_2=YES** |
| **27.** | fa_1a | What else made it easier for you to exercise or perform physical activity today? | Text answer | **IF fa_1=6** |
| **28.** | kontxd _3 | Today it was only up to me how physically active I was. | (0)“Not at all” – (100) “Very much so” |  |
| **29.** | kontxd_4 | Do you intend to exercise or be physically active tomorrow? | (0)“NO”, (1)“YES” |  |

**Note.***marks exclusive options

**II.SELF-INITIATED PHYSICAL ACTIVITY SURVEY**

| **Item order#** | **Variable** | **Item** | **Response Format** | **Branching** |
| --- | --- | --- | --- | --- |
|  | | | *Slider with word anchors* |  |
| **1.** | fs_1 | How do you feel right now? | 0) “Very bad” – (100) “Very good” |  |
| **2.** | čas_1 | How long ago was your episode of physical activity? | Minutes (scrolled) |  |
| **3.** | pa_2 | What type of physical activity did you do? | *Checkbox (selection)*  (1) slow wlaking  (2) fast walking  (3) jogging or running  (4) bicycling  (5) yard word  (6) heavy household chores (e.g., vacuuming)  (7) other | **IF pa_1=YES** |
| **4.** | pa_2a | What other type of physical activity did you do? | Text answer | **IF pa_2 =7** |
| **5.** | pa_3 | How long did you do this activity for? | Minutes (scrolled) | **IF pa_1=YES** |
| **6.** | pa_4 | How physically demanding was this activity? | (0) “Very light” – (100) “Very intense” | **IF pa_1=YES** |
| **7.** | pa_5 | Was this activity planned? | (0)“NO”  (1)“YES” | **IF pa_1=YES** |
| **8.** | pa_6 | Where did you do this activity? | (1) at home  (2) outside in nature  (3) outside in a park  (4) in fitness center/sports club  (5) in the street  (6) elsewhere | **IF pa_1=YES** |
| **9.** | pa_6a | Where else did you do this activity? | Text answer | **IF pa_6=6** |
| **10.** | pa_7 | Who did you do this activity with? (select all that applies) | (1) *with noone, alone  (2) with a partner  (3) with friends  (4) with family  (5) with coworkers  (6) with someone else | **IF pa_1=YES** |
| **11.** | bol_1 | Do you feel any pain right now? | (0)“NO”, (1)“YES” |  |
| **12.** | bol_1a | How intense is your pain? | (0) “Little, I am coping well” – (100) “Very, I am not coping well” | **IF bol_1=YES** |

**Note.***marks exclusive answer options

**III.CONTEXT TRIGGERED PHYSICAL ACTIVITY SURVEY**

| **Item order#** | **Variable** | **Item** | **Response Format** | **Branching** |
| --- | --- | --- | --- | --- |
|  | | | *Slider with word anchors* |  |
| **1.** | fs_1 | How do you feel right now? | 0) “Very bad” – (100) “Very good” |  |
| **2.** | pa_1 | Did you do any physical activity in the past hour? | (0)“NO”  (1)“YES” |  |
| **3.** | pa_2 | What type of physical activity did you do? | *Checkbox (selection)*  (1) slow wlaking  (2) fast walking  (3) jogging or running  (4) bicycling  (5) yard word  (6) heavy household chores (e.g., vacuuming)  (7) other | **IF pa_1=YES** |
| **4.** | pa_2a | What other type of physical activity did you do? | Text answer | **IF pa_2 =7** |
| **5.** | pa_3 | How long did you do this activity for? | Minutes (scrolled) | **IF pa_1=YES** |
| **6.** | pa_4 | How physically demanding was this activity? | (0) “Very light” – (100) “Very intense” | **IF pa_1=YES** |
| **7.** | pa_5 | Was this activity planned? | (0)“NO”  (1)“YES” | **IF pa_1=YES** |
| **8.** | pa_6 | Where did you do this activity? | (1) at home  (2) outside in nature  (3) outside in a park  (4) in fitness center/sports club  (5) in the street  (6) elsewhere | **IF pa_1=YES** |
| **9.** | pa_6a | Where else did you do this activity? | Text answer | **IF pa_6=6** |
| **10.** | pa_7 | Who did you do this activity with? (select all that applies) | (1) *with noone, alone  (2) with a partner  (3) with friends  (4) with family  (5) with coworkers  (6) with someone else | **IF pa_1=YES** |
| **11.** | bol_1 | Do you feel any pain right now? | (0)“NO”, (1)“YES” |  |
| **12.** | bol_1a | How intense is your pain? | (0) “Little, I am coping well” – (100) “Very, I am not coping well” | **IF bol_1=YES** |

**Note.***marks exclusive answer options
